# Confocal laser endomicroscopy improves diagnosis of cholangiocarcinoma: a case report and review

**DOI:** 10.1515/biol-2025-1269

**Published:** 2026-01-26

**Authors:** Zewen Xu, Yongrong Li, Liwei Dong, Chaochao Chen, Wenwen Wang, Zhoutao He, Cheng Lan

**Affiliations:** Department of Gastroenterology, Hainan General Hospital, Hainan Affiliated Hospital of Hainan Medical University, Haikou, 570311, China; Department of Radiology, Hainan General Hospital, Hainan Affiliated Hospital of Hainan Medical University, Haikou, 570311, China

**Keywords:** cholangiocarcinoma, confocal laser endomicroscopy, malignant tumor, bile duct stricture, diagnosis

## Abstract

Cholangiocarcinoma (CCA), a life-threatening malignancy with a poor prognosis, remains diagnostically challenging due to the limited sensitivity and specificity of traditional imaging in differentiating malignant from benign bile duct strictures. This case report and literature review explore the potential of confocal laser endomicroscopy (CLE), an emerging technology for real-time *in vivo* microscopic imaging, to address this gap. We present the case of a 64-year-old male presenting with scleral icterus, choluria, progressive jaundice, hyperbilirubinemia, and radiologically confirmed biliary dilation. During endoscopic retrograde cholangiopancreatography, CLE revealed intraoperative imaging features suggestive of CCA – a finding subsequently confirmed by postoperative histopathology. Together with supporting literature, this case underscores the clinical utility of CLE by providing high-resolution, real-time visualization of characteristic features, thereby directly aiding in the diagnosis of indeterminate biliary strictures.

## 1 Introduction

Cholangiocarcinoma (CCA), a malignancy arising from the biliary epithelium, is the most prevalent form of biliary tract cancer [1]. Its global incidence and mortality have been steadily increasing, imposing a particularly substantial disease burden on Asian populations [2]. Despite progress in diagnostic modalities, the early detection of CCA remains challenging owing to its nonspecific clinical presentation, highly aggressive nature, and the limited sensitivity of available imaging and serological biomarkers. As a result, approximately 60–70 % of patients are diagnosed at an advanced stage, thereby forfeiting the opportunity for curative surgical intervention [3]. For these patients, palliative systemic chemotherapy is the mainstay of treatment; however, the prognosis is exceedingly poor, with a median survival of less than one year and a five-year survival rate of merely 5 % [4]. Therefore, early detection and diagnosis are paramount for improving patient outcomes. This underscores the pressing clinical need for novel technologies that can accurately distinguish biliary strictures and improve early diagnosis rates.

Conventional imaging modalities, such as color Doppler ultrasonography, continue to play an essential role in the initial evaluation of hepatobiliary diseases due to their non-invasive nature, cost-effectiveness, and widespread availability [5]. These techniques are valuable for assessing hepatic parenchyma, vascular flow, and detecting larger focal lesions. However, their resolution is insufficient for the precise characterization of subtle mucosal abnormalities or early neoplastic changes confined to the biliary epithelium. To address this diagnostic gap, technologies capable of visualizing the biliary mucosa at a microscopic level have therefore been developed.

Confocal laser endomicroscopy (CLE) is an advanced imaging technique that provides real-time, cellular-level resolution during endoscopy, offering a novel approach for the *in vivo* diagnosis of biliary tract diseases [6]. Although preliminary studies have shown promise, its diagnostic performance and incremental value in routine clinical practice necessitate further investigation with larger case series. This case report elucidates the CLE procedure, its imaging features, and its decisive role in diagnosing indeterminate bile duct strictures through a pathologically confirmed case of CCA. By comparing CLE findings with conventional imaging results, this report aims to illustrate the added diagnostic value of CLE and support its adoption in the evaluation of indeterminate biliary strictures.

## 2 Case report

### 2.1 Patient information

A 64-year-old man was admitted with a one-month history of progressive jaundice, dark urine, and anorexia. He also reported occasional dizziness. Since symptom onset, he had experienced lethargy, insomnia, and an unintentional weight loss of 7 kg. His bowel habits were normal.

His past medical history was significant for occupational trauma 10 years ago and hypertension diagnosed one year ago. Although he adhered to antihypertensive medication, his blood pressure was poorly controlled, with historical systolic readings exceeding 200 mmHg and current fluctuations between 140 and 160 mmHg. Notably, his admission laboratory panel was negative for hepatitis B surface antigen (HBsAg), anti-HCV antibody, and SARS-CoV-2 antigen, effectively ruling out active infection with hepatitis B, hepatitis C, or COVID-19.

**Informed consent:** Informed consent has been obtained from all individuals included in this study.

**Ethical approval:** The research related to human use has been complied with all the relevant national regulations, institutional policies and in accordance with the tenets of the Helsinki Declaration, and has been approved by the Medical Ethics Committee of Hainan General Hospital (Approval No.: Med-Eth-Re [2025] 630).

### 2.2 Laboratory investigations

Progressive Obstructive Jaundice: Total bilirubin increased from 97.75 μmol/L (February 5) to 192.43 μmol/L (February 19), predominantly direct.

Tumor Marker Profile: Carbohydrate antigen 19-9 (CA19-9) was elevated to 293.47 U/mL (February 19). Lactate dehydrogenase (LDH) was within normal range (155.1 U/L).

Inflammation and Hematology: C-reactive protein (CRP) was mildly elevated (5.69 mg/L on February 19, rising to 7.74 mg/L by February 26). Complete blood count showed mild anemia (hemoglobin 127 g/L) with normal white cell and platelet counts.

### 2.3 Imaging examinations

A contrast-enhanced computed tomography (CT) scan of the upper abdomen, performed on the same day, demonstrated dilation of the intra- and extrahepatic bile ducts. Furthermore, the walls of the cystic duct and common bile duct appeared thickened and exhibited enhancement, with a maximum thickness of approximately 0.8 cm. The initial radiological impression raised suspicion for either a neoplastic process (e.g., tumor) or inflammation.

Subsequent magnetic resonance imaging (MRI) was pursued for further characterization. An upper abdominal MRI with Magnetic Resonance Cholangiopancreatography (MRCP) on February 20, 2024, confirmed a segmental stenosis in the upper common bile duct, causing significant upstream biliary dilation. The radiologist recommended an enhanced scan for a definitive diagnosis.

Accordingly, a contrast-enhanced MRI of the upper abdomen was performed on February 23, 2024. Compared to the previous non-enhanced MRI (February 19, 2024), this study identified a soft tissue lesion at the pancreatic segment of the common bile duct, which was responsible for the biliary obstruction. The definitive imaging conclusion favored a neoplastic lesion, with CCA being the primary differential diagnosis.

### 2.4 Endoscopic examination and CLE procedure

The patient was placed in a prone position. A duodenoscope was introduced and advanced successfully into the second portion of the duodenum. The major duodenal papilla appeared granular and measured approximately 1.0 × 1.0 × 1.0 cm. Selective cannulation of the bile duct was achieved. Initial attempts to advance a guidewire were met with resistance beyond the mid-common bile duct. Subsequently, a hydrophilic guidewire was employed, which was successfully advanced into the intrahepatic bile ducts.

**Figure 1:**
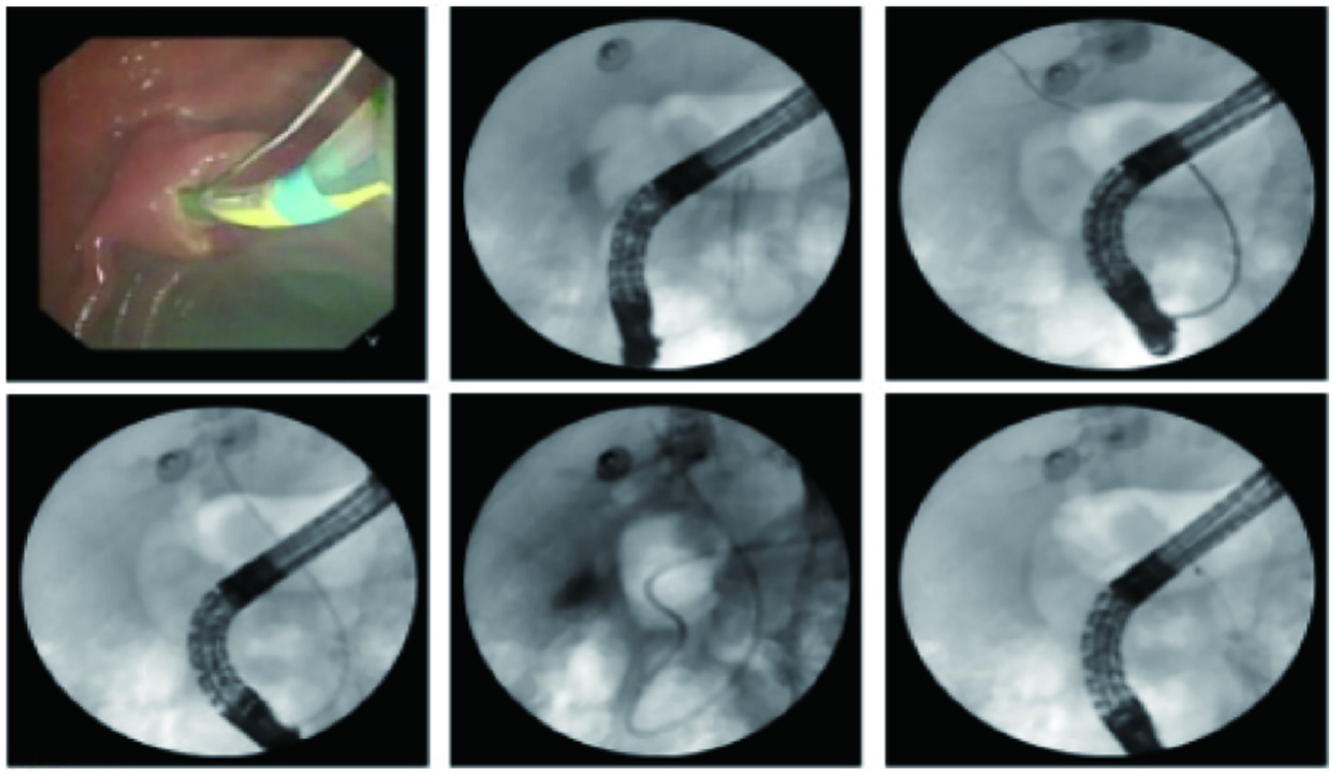
Narrowing of the middle segment of the common bile duct and dilation of the intrahepatic bile duct.

A cholangiogram was then obtained by injecting 10 mL of Iopamidol contrast medium through a sphincterotome. Fluoroscopic imaging revealed a segmental stenosis, approximately 6 cm in length, in the mid-common bile duct. Significant dilation of the common bile duct (approximately 2.0 cm in diameter) and intrahepatic bile ducts was noted proximal to the stricture (Figure 1).

**Figure 2:**
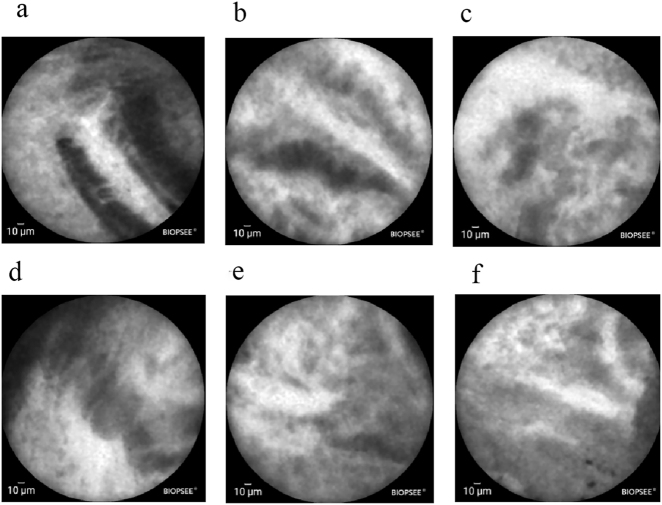
Visualized biopsy diagnostic image (a–d) exhibit widened dark bands (>40 μm wide), along with dark clusters and black masses of the epithelium. (e–f) Reveal bright bands that are twisted, thickened, and wide, exhibiting a width greater than 20 μm.

Following intravenous administration of 10 mL of 20 % fluorescein sodium, a single-use cholangioscopic CLE probe (Jingwei Shida, BIOPSEE^®^, 0.93 mm diameter) was introduced over the guidewire to the target site. The lesion was systematically scanned, and the acquired images were interpreted in real-time according to the Paris Classification criteria (Figure 2).

Subsequently, an endoscopic sphincterotomy of approximately 0.5 cm was performed. The CLE probe was then used to guide tissue sampling by providing real-time, *in situ* visualization of the lesion. A targeted biopsy was obtained from the area with the most characteristic CLE features using biopsy forceps, and the specimen was sent for histopathological analysis.

Finally, a nasobiliary drainage tube was placed over the guidewire and secured. The procedure was concluded successfully, and the patient tolerated it well without any immediate complications.

### 2.5 Histopathological analysis

**Figure 3:**
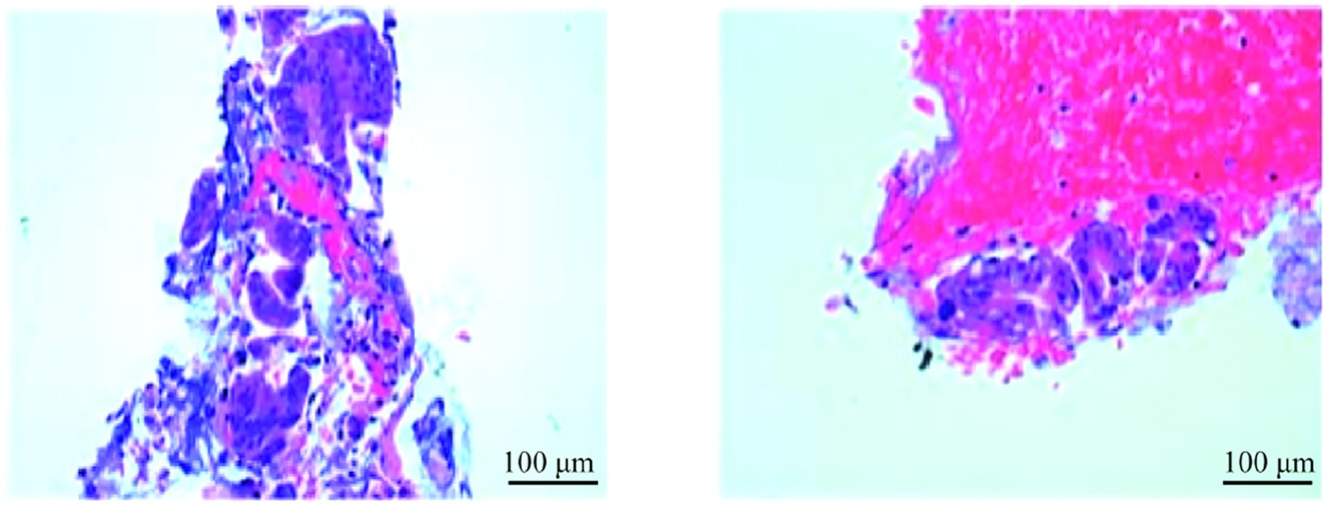
Histopathological image of the biopsy specimen (HE staining). The image shows a cluster of atypical epithelial cells consistent with adenocarcinoma, characterized by abundant cytoplasm, a high nuclear-to-cytoplasmic (N/C) ratio, and irregular nuclear contours. Mitotic activity is not evident in this field. Original magnification: ×100.

The biopsy specimens obtained during the procedure were fixed in formalin, embedded in paraffin, and sectioned. Sections (4-μm thick) were stained with hematoxylin and eosin (HE) following the standard clinical protocol at our institution’s Department of Pathology. All histopathological slides were examined by an experienced pathologist using a conventional light microscope. The representative photomicrograph in Figure 3 was taken at a magnification of ×100.

### 2.6 Subsequent management

The patient was transferred to the Department of Hepatobiliary Surgery and underwent pancreaticoduodenectomy, cholecystectomy, regional lymph node dissection, and cholangioplasty. The patient’s condition stabilized postoperatively, and he was advised to attend regular follow-up appointments after discharge.

## 3 Discussion

The accurate diagnosis of indeterminate bile duct strictures remains a significant clinical challenge. This case of pathologically confirmed CCA illustrates that the real-time, *in vivo* cytology offered by CLE effectively narrows the diagnostic gap between imaging and tissue diagnosis.

CCA is a leading cause of malignant biliary strictures, making accurate differentiation from benign conditions critical. Current preoperative diagnosis relies heavily on multimodal imaging. However, these modalities have inherent limitations in providing a definitive qualitative diagnosis. Ultrasound, valued for being non-invasive, convenient, and radiation-free, serves as a primary screening tool for detecting biliary obstruction and intrahepatic masses [7]. Nevertheless, its sensitivity for small or infiltrative CCA is suboptimal, and its accuracy in tumor sizing is operator-dependent [8]. CT is the standard for tumor staging and characterization, offering clear delineation of a lesion’s anatomical location, size, and relationship to surrounding structures [9]. However, one study reported a diagnostic accuracy of 89.42 % for CT, corresponding to an error rate of approximately 10 % [10]. MRI with MRCP is considered the preferred combination due to its superior soft-tissue resolution, which allows for precise assessment of tumor morphology and vascular involvement [11]. Despite this, one study demonstrated a diagnostic sensitivity of only 59.6 % against a high specificity of 95.7 %, indicating a concerningly high rate of missed diagnoses [12]. Furthermore, the diagnostic performance of positron emission tomography for infiltrative tumors and extrahepatic CCA remains inconsistent [13].

The limitations of these advanced imaging techniques were evident in the present case. While contrast-enhanced CT revealed bile duct wall thickening and dilation, and MRI/MRCP identified a stricture in the upper common bile duct, neither could conclusively determine its benign or malignant nature. It is precisely this common diagnostic blind spot that underscores the urgent clinical need for novel technologies like CLE, which enables *in vivo* histological assessment. This case provides a clear rationale for evaluating the additional diagnostic value of CLE in bridging this critical gap.

To achieve the goal of *in vivo* histological evaluation, CLE provides a unique technical solution. First, CLE provides real-time, high-magnification (e.g., 1,000×) visualization of microscopic tissue architecture, revealing cellular and microvascular details [14], 15]. This is particularly crucial when high-quality physical specimens are difficult to obtain, such as in fibrotic or rigid strictures. Second, CLE enables real-time diagnostic assessment. By analyzing characteristic features such as the width of bright and dark bands, dark clusters, and epithelial structures, it allows endoscopists to provide immediate diagnostic interpretation during the procedure. This real-time feedback can directly guide clinical decisions, such as whether to proceed with surgery, which significantly shortens the diagnostic process. In the present case, when traditional imaging indicated an indeterminate stricture, the microscopic patterns observed with CLE were highly suggestive of malignancy. This finding not only increased diagnostic confidence but also provided crucial targeting for the subsequent biopsy.

The technical advantages of CLE are fully realized in its integration with ERCP. Although ERCP is the first-line method for diagnosing and treating bile duct obstruction, its guided tissue sampling techniques (e.g., biopsy and brush cytology) exhibit low sensitivity (48 % and 45 %, respectively), which continues to pose significant challenges in diagnosing indeterminate bile duct strictures [16]. The integration of CLE with ERCP directly addresses this critical limitation. In the present case, the real-time observation of cellular microstructures via CLE during an ERCP procedure enabled the successful diagnosis of malignant biliary stenosis. Numerous studies support the superiority of this combined approach. For instance, a multicenter study demonstrated that the diagnostic accuracy of ERCP with pCLE is significantly higher than that of ERCP with tissue sampling alone (90 % vs. 73 %) [17]. Furthermore, another report indicates that this strategy achieves a sensitivity of up to 98 % for detecting malignant strictures, far exceeding the 45 % sensitivity of conventional ERCP-guided sampling [18].

The successful integration of CLE into the ERCP platform exemplifies its high diagnostic performance. This is robustly supported by a growing body of evidence demonstrating CLE’s consistently high sensitivity for biliary tumors. A retrospective study initially highlighted the diagnostic power of CLE, showing that probe-based CLE (pCLE) achieved a sensitivity and negative predictive value of 100 % for distinguishing biliary tract tumors and markedly outperformed conventional tissue sampling [19]. This finding was subsequently validated by a prospective, multicenter international study, which established the comprehensive superiority of pCLE. Specifically, pCLE demonstrated significantly higher sensitivity (89 % vs. 56 %) and overall accuracy (82 % vs. 72 %) for diagnosing CCA compared to standard histological sampling alone [20]. More recently, a multicenter prospective trial reaffirmed the high sensitivity of pCLE (85.7 %) through a direct, contemporaneous comparison with conventional techniques, demonstrating its substantial sensitivity advantage over fluorescence *in situ* hybridization (60 %), forceps biopsy (50 %), and brush cytology (14.3 %) [21].

This conclusion is supported by high-level evidence, including meta-analyses of multiple studies. One meta-analysis, pooling data from eight studies, reported a pooled sensitivity of 90 % and specificity of 75 % [22]. Furthermore, a separate meta-analysis directly comparing pCLE with tissue sampling during ERCP demonstrated that pCLE has a significantly superior diagnostic odds ratio and a larger area under the SROC curve [14]. In summary, the present case, consistent with robust cumulative evidence, strongly indicates that CLE addresses the critical limitations of traditional diagnostics through real-time, *in vivo* histology and emerges as a transformative tool for the management of indeterminate biliary strictures.

Furthermore, the laboratory profile exhibited a distinct pattern. Serum LDH was normal, contrasting with its typical elevation in advanced tumors associated with high burden and poor prognosis [23]. Conversely, CA19-9 was markedly elevated, strongly supporting a biliary tract origin. A concurrent mild increase in C-reactive protein suggested tumor-related inflammation. This dissociation – normal LDH alongside elevated CA19-9 – provides a useful diagnostic clue to differentiate CCA from more aggressive malignancies that commonly drive significant LDH elevation, underscoring the value of a multiplexed interpretation of tumor markers.

However, it is crucial to acknowledge that the core challenge in diagnosing CCA extends beyond technical limitations to encompass its distinct biological behavior. A recent multi-omics study demonstrated that bile acid–driven accumulation of sialic acid on the surface of cancer cells constitutes a key molecular mechanism underlying their high metastatic potential [24]. Such molecular-level alterations may contribute to atypical tumor presentations in conventional imaging and histopathological assessment, thereby compounding the difficulties of early detection and precise staging.

Building on this mechanistic insight, future strategies to enhance diagnostic and staging accuracy are likely to follow a multimodal path. One promising direction involves developing novel detection tools – such as molecular imaging probes or liquid biopsy assays – targeting specific molecular pathways such as sialic acid metabolism. These approaches could provide complementary functional and molecular data beyond traditional morphology. Concurrently, the refinement of existing morphological diagnostic techniques remains essential. For instance, the CLE features observed in the present case – including irregular dark lumenal structures and thickened white bands – align closely with the diagnostic criteria for CCA established in prior pivotal studies [25], 26]. This alignment validates the imaging biomarkers and corroborates the high diagnostic yield of CLE in real-time assessment of indeterminate biliary strictures reported in those series.

However, CLE technology is subject to several limitations. First, CLE image interpretation is inherently subjective and requires extensive, specialized training to master, which can lead to significant inter-observer variability. Second, benign conditions such as inflammatory reactions or reactive hyperplasia can produce malignant-like image features (e.g., thickened white bands), potentially leading to false-positive interpretations. Furthermore, although CLE, imaging, and HE staining yielded highly concordant results, this retrospective study lacked immunohistochemical (IHC) validation due to the clinical constraints at the time. Future prospective studies should incorporate IHC as per current guidelines [27], which would provide a gold-standard diagnostic control and enable the correlation of CLE features with tumor molecular phenotypes. Finally, the generalizability of our findings is limited by the nature of this study as a single-center case report, which provides a low level of evidence. To address these challenges, large-scale, prospective, multicenter studies are warranted to validate the efficacy of CLE and standardize its operational protocols. Concurrently, the integration of artificial intelligence for assisted diagnosis represents a promising avenue to reduce interpretive subjectivity and improve both the consistency and accuracy of image analysis.

In summary, CLE bridges the diagnostic gap between radiologic imaging and histopathology for indeterminate biliary strictures by providing real-time, *in vivo* microscopic visualization of the mucosal architecture. Despite challenges such as operator dependence and cost, it promises to be an indispensable adjunct within future multidisciplinary diagnostic strategies.
